# Beyond Decompression: Successes and Failures in the Modern Care of Degenerative Cervical Myelopathy

**DOI:** 10.3390/neurolint18050090

**Published:** 2026-05-13

**Authors:** Andreas K. Demetriades

**Affiliations:** 1 Edinburgh Spinal Surgery Outcome Studies Group, Department of Neurosurgery, Royal Infirmary Edinburgh, NHS Lothian, Edinburgh EH16 4SA, UK; andreas.demetriades@gmail.com; 2 Department of Neurosurgery, Leiden University Medical Centre, 2300 RC Leiden, The Netherlands

Surgical mastery has transformed treatment, but diagnosis, neuroprotection, and recovery remain the field’s next frontier.

There are few conditions in medicine that so clearly expose the distance between what we can do and what we can truly achieve as degenerative cervical myelopathy (DCM). It is a disorder in which modern surgery can relieve pressure with extraordinary precision, yet the injured spinal cord often remains biologically vulnerable, difficult to protect, and only partially responsive to intervention [1]. The field has advanced with real force; imaging is sharper, operative planning is more disciplined, and decompression is safer than ever before. And yet the central paradox remains unresolved. We have become highly accomplished in correcting the anatomy of disease, but we are still far less effective at influencing the biology that determines whether patients recover [2].

The progress on the surgical side is substantial and should be recognized as such. Contemporary management is grounded in more rigorous evidence, the more refined selection of approach, and a more nuanced appreciation of spinal alignment, number of involved levels, and the distribution of compression. Anterior, posterior, and combined procedures are now chosen with far greater precision than in earlier eras, making surgery not only more effective, but more rational. MRI has also transformed the diagnostic landscape, allowing clinicians to move beyond the mere identification of stenosis and to infer something of the cord’s condition itself. Signal change on T2-weighted images, and especially T1 hypointensity when present, helps shape expectations and prognosis [3,4,5,6]. Intraoperative neurophysiological monitoring adds another layer of safety, offering real-time warning and reassurance during the most delicate stages of decompression [7]. These are genuine achievements. They represent a real maturation of the field.

Yet the story of myelopathy cannot be told through surgical success alone, because some of the most consequential failures occur before the patient ever reaches the operating theatre. The first is delay. Degenerative cervical myelopathy is a master of misdirection. Its early manifestations are often subtle, incomplete, and easily attributed to far more common explanations: peripheral nerve entrapment, age-related slowing, arthritis, lumbar disease, or nonspecific clumsiness. The result is a diagnostic journey that is too often prolonged, fragmented, and frustrating. For many patients, months pass before the possibility of cord disease is seriously entertained, and sometimes years before it is confirmed [8,9]. In a disorder where time matters so deeply, delay is not a neutral event—it is a biological one. The longer the cord remains compromised, the less reversible the injury is likely to become.

The second failure is more fundamental still: we remain without a truly effective biological treatment for the disease itself. Surgery addresses the compression, but not the full cascade of injury it unleashes. Chronic ischemia, inflammation, excitotoxicity, demyelination, axonal injury, and impaired repair all contribute to the pathology of myelopathy. We can remove the structural insult, but we cannot yet reliably protect the cord from the consequences of that insult, nor can we confidently alter the course of recovery once decompression has occurred. This is the field’s most important translational gap. In many areas of medicine, treatment has moved from simply removing the lesion to modifying the disease. In myelopathy, that transition has not yet been achieved [10,11].

A third limitation lies in the way recovery is often conceived. Too frequently, postoperative care is framed as a period of passive observation, as though decompression itself were sufficient and biology would take care of the rest. But recovery after myelopathy is often slow, partial, and uneven. Some patients improve meaningfully; others stabilize but remain impaired; and many continue to struggle with gait disturbance, hand dysfunction, sensory symptoms, or fatigue long after surgery. Rehabilitation, despite its obvious relevance, remains inconsistently developed and insufficiently standardized as a specific therapeutic domain. That is a serious omission. Recovery is not an afterthought: it is the outcome by which intervention is ultimately judged, and it deserves the same seriousness that the field has long given to operative technique [12,13].

It is against this background that RECEDE-Myelopathy becomes especially noteworthy. The trial represents more than a research project; it reflects a change in the field’s imagination. By evaluating ibudilast in degenerative cervical myelopathy, it asks whether pharmacological intervention can begin to address the biological dimension of cord injury rather than only the mechanical one [14]. That question matters. If such a therapy can influence neuroinflammation, stabilize injury, or support recovery around the time of decompression, then the field will have taken a decisive step beyond surgery alone. Even before results are known, the significance of the study lies in its ambition: it acknowledges that myelopathy may require more than a scalpel.

What comes next must be broader than any single trial or technique.

First, diagnosis has to become earlier, sharper, and more widely shared as a clinical responsibility. The burden cannot rest only with specialists. General practitioners, physiotherapists, neurologists, and other first-contact clinicians must be equipped to recognize the subtle signs of cord disease sooner, so that imaging and referral occur before disability becomes entrenched [8,9,15,16]. Second, the search for neuroprotection must continue with more urgency and more discipline. A field that understands the biological complexity of myelopathy should not be satisfied with decompression as its only active tool. Third, recovery must be made intentional. Rehabilitation pathways should be structured, tested, and tailored to the functional challenges that myelopathy leaves behind [17]. If we want better outcomes, we must stop treating recovery as a by-product and begin treating it as a therapeutic target.

The real measure of progress in myelopathy will not be how elegantly we operate, but how completely we restore. Surgery has brought the field far, and it remains indispensable, but the future cannot belong to mechanics alone. It must belong to earlier recognition, to biological insight, and to recovery that is actively built rather than passively awaited. That is the next frontier: not merely to relieve pressure on the spinal cord, but to preserve what remains, protect what is threatened, and restore what has been lost.

## Data Availability

The original contributions presented in this study are included in the article. Further inquiries can be directed to the corresponding author.
